# Supplementary material for: Breaking the trade-off between fast control and long lifetime of a superconducting qubit
Source: Nat Commun. 2020 Jul 23;11:3683. doi: 10.1038/s41467-020-17511-y (PMC7378077; doi:10.1038/s41467-020-17511-y)
Supplement: Supplementary file 1 — Supplementary Information [file 41467_2020_17511_MOESM1_ESM.pdf]

Supplementary Information for "Breaking the trade-off between fast control and long lifetime of a superconducting qubit"

S. Kono[1], K. Koshino[2], D. Lachance-Quirion[3,4], A. F. van Loo[1], Y. Tabuchi[3], A. Noguchi[5,6], and Y. Nakamura[1,3]

[1]*Center for Emergent Matter Science (CEMS), RIKEN, Wako, Saitama 351-0198, Japan*
[2]*College of Liberal Arts and Sciences, Tokyo Medical and Dental University, Ichikawa, Chiba 272-0827, Japan*
[3]*Research Center for Advanced Science and Technology (RCAST), The University of Tokyo, Meguro-ku, Tokyo 153-8904, Japan*
[4]*Institut quantique and Département de physique, Université de Sherbrooke, Sherbrooke, Québec, J1K 2R1, Canada*
[5]*Komaba Institute for Science (KIS), The University of Tokyo, Meguro-ku, Tokyo, 153-8902, Japan and*
[6]*PRESTO, Japan Science and Technology Agency, Kawaguchi-shi, Saitama 332-0012, Japan*

Supplementary Note 1: Sample information

An optical microscope image of a nominally identical sample to the one used in this work is shown in Supplementary Figures 1a–f. Three qubits are dispersively coupled to their respective readout resonators, which are connected to a readout line via a Purcell filter. The transmon qubit, which is coupled to a control coplanar waveguide, is used as a data qubit in the main experiments (see Supplementary Figures 1d and e). A Josephson quantum filter (JQF), which is a frequency-tunable transmon qubit with a capacitor composed of a single electrode and the ground plane, is strongly coupled to the control line approximately half a wavelength apart from the data qubit (see Supplementary Figures 1b and c).

To fabricate the superconducting circuit, a 200-nm thick Nb film on a high-resistivity (>10 kΩ-cm) silicon substrate is patterned with photolithography followed by CF_4 plasma etching to form the larger structures, such as the transmon pads, JQF pad, readout resonators, Purcell filter, and coplanar waveguides. The magnetic-flux-trapping holes are also fabricated on the ground plane in this step. Following O_2 plasma ashing and HF cleaning, the $Al/AlO_x/Al$ Josephson junctions are fabricated with electron-beam lithography followed by electron-beam evaporation using a bridgeless process. While the fixed frequency data qubit contains a single Josephson junction, the JQF contains an asymmetric SQUID in order to be able to tune its frequency to be resonant with the data qubit [1]. The 300-nm thick Al air-bridges with heights of about 3 μm (see Supplementary Figure 1f) are fabricated with a four-step process: photolithography followed by electron-beam evaporation and another photolithography followed by Al etching. Together with the Al air-bridges, a frame and bonding pads made of Al are fabricated to make good contacts with Al bonding wires. After dicing, the device is carefully cleaned with NMP and with O_2 plasma ashing.

As listed in Supplementary Table I, we determine the system parameters by comparing the experimental data with theoretical models in Supplementary Note 3.

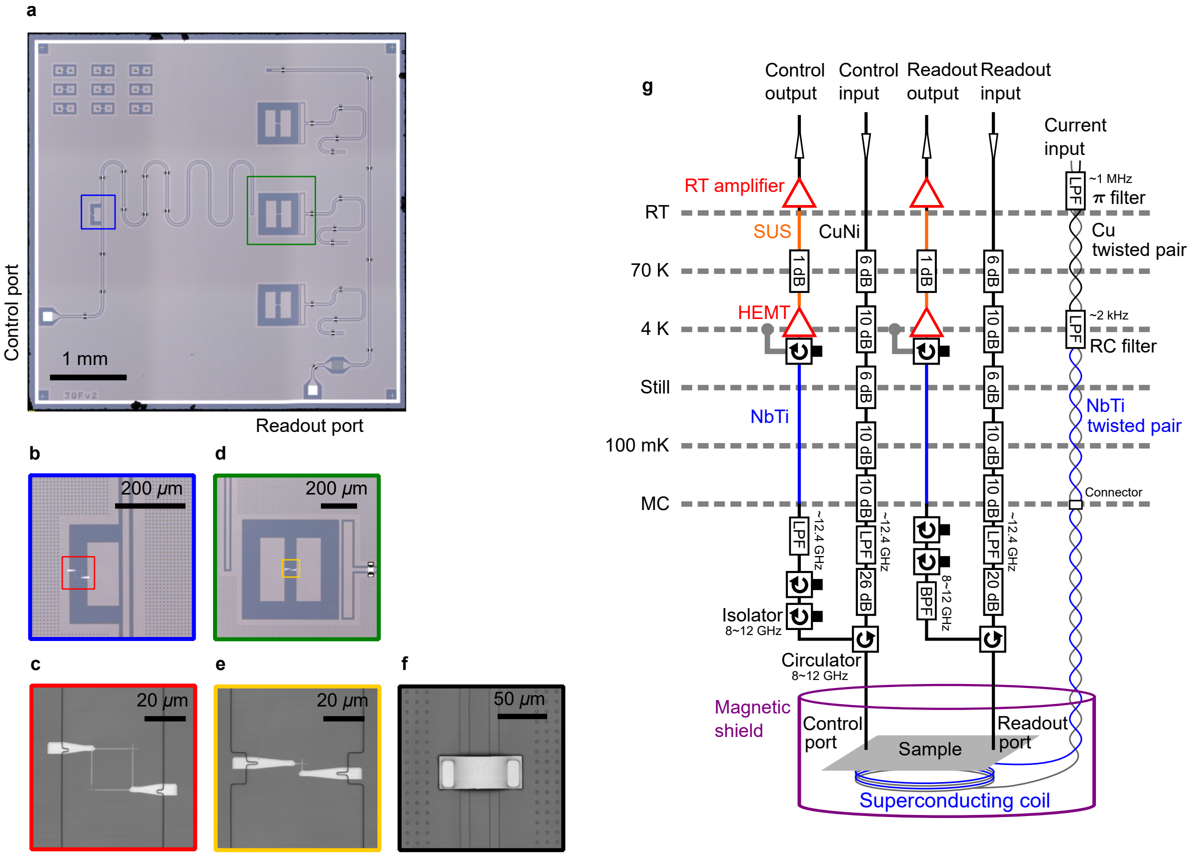

Supplementary Figure 1: Sample and experimental setup. **a**, Optical microscope image of the entire sample. **b,c**, JQF and its asymmetric SQUID. **d,e**, Data qubit and its single Josephson junction. **f**, An air-bridge. **g**, Schematic of the experimental setup.

Supplementary Note 2: Experimental setup

The experimental setup is shown in Supplementary Figure 1g. The superconducting circuit is mounted in a sample holder placed in a magnetic shield at the base temperature stage ($\sim$ 50 mK) of a dilution refrigerator. A magnetic field is applied to the SQUID of the JQF by a superconducting coil placed around the chip. Radio-frequency noise through the flux-bias line is removed by low-pass filters: a π filter at room temperature and an RC filter at the 4 K stage. The microwave input line is highly-attenuated and filtered to decrease noise from room temperature. A cryogenic HEMT amplifier and a room-temperature low-noise amplifier are installed in the microwave output line to measure weak microwave signals. Several circulators and filters are installed in the output line to reduce the backward noise from the amplifiers.

The readout resonator is characterized via the readout line using reflection measurements in the frequency domain. The qubit is characterized using two-tone spectroscopy. Furthermore, the qubit and JQF are further characterized via the control line by the reflection measurements in the frequency domain. Then, the qubit state is controlled by applying resonant microwave pulses via the control line, and is read out by measuring the dispersive shift of the readout resonator via the readout line in the time domain. We study the effect of the JQF on the qubit control by measuring the relaxation and Rabi oscillation of the qubit in both cases when the JQF is in or out of resonance with the qubit.

The microwave pulses for control and readout are generated by mixing low-frequency pulse signals with continuous microwaves. The low-frequency signals are generated at 1-GHz sampling rates with digital-analog converters. The readout pulses are down-converted at the mixer using a continuous microwave signal phase-locked with the one used for the pulse generation and are measured at a 1-GHz sampling rate by an analog-digital converter.

Supplementary Table I: System parameters. Note that λ_q ($\approx$ 15 mm) is the wavelength at the qubit frequency.

Qubit	Value	
Resonance frequency, $\omega_\mathrm{q}/2\pi$	8.002 GHz	
Anharmonicity, $\alpha_\mathrm{q}/2\pi$	-0.398 GHz	
External coupling rate, $\gamma_\mathrm{ex}^\mathrm{q}/2\pi$	123 kHz	
Intrinsic loss rate, $\gamma_\mathrm{in}^\mathrm{q}/2\pi$	16 kHz	
Pure dephasing rate, $\gamma_\phi^\mathrm{q}/2\pi$	6 kHz	
Intrinsic thermal quanta, n_th^q	0.29	
	w/o JQF	w/ JQF
Relaxation time, T_1	1.1 μs	5.2 μs
Coherence time, T_2^E	2.3 μs	7.3 μs
Coherence time, T_2^*	1.7 μs	3.8 μs
Thermal population, p_th^q	0.028	0.16

JQF	Value
Resonance frequency, $\omega_\mathrm{f}/2\pi$	6.3$-$8.5 GHz
Anharmonicity, $\alpha_\mathrm{f}/2\pi$	-0.387 GHz
External coupling rate, $\gamma_\mathrm{ex}^\mathrm{f}/2\pi$	113 MHz
Intrinsic loss rate, $\gamma_\mathrm{in}^\mathrm{f}/2\pi$	3 MHz
Pure dephasing rate, $\gamma_\phi^\mathrm{f}/2\pi$	$\lesssim$ 100 kHz
Intrinsic thermal quanta, n_th^f	≈ 0
Separation between qubit and JQF, d	$0.526\lambda_\mathrm{q}$

Readout resonator	Value
Resonance frequency, $\omega_\mathrm{r}/2\pi$	10.1564 GHz
External coupling rate, $\kappa_\mathrm{ex}/2\pi$	2.152 MHz
Intrinsic loss rate, $\kappa_\mathrm{in}/2\pi$	0.015 MHz
Dispersive frequency shift, $2\chi/2\pi$	1.870 MHz

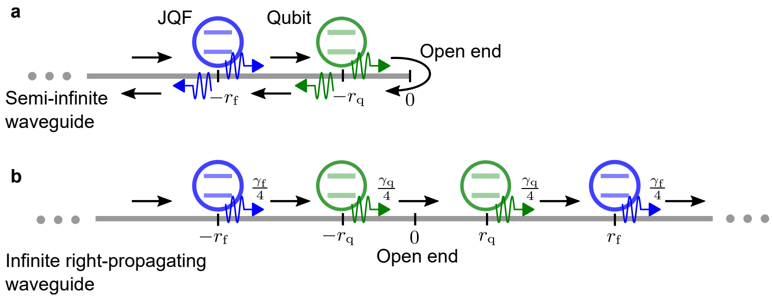

Supplementary Figure 2: Schematic of the theoretical model. **a**, System where a qubit and a JQF are coupled to a semi-infinite waveguide. **b**, System where a qubit and a JQF are coupled twice to the right-propagating modes of an infinite waveguide.

Supplementary Note 3: Theoretical model

To numerically simulate the experimental data, we generalize the theoretical model discussed in the reference [2], i.e. we consider a system where two transmon qubits (a data qubit and a JQF) with different resonance frequencies are coupled to a semi-infinite waveguide, as shown in Supplementary Figure 2a. In this system, a propagating field interacts with each qubit twice at different points before and after the reflection by the open end. Therefore, it can be modeled as a system where two qubits are coupled at two different points to an infinite waveguide containing only right-propagating modes, as shown in Supplementary Figure 2b. The qubit (JQF) is coupled to the waveguide at positions $-r_\mathrm{q}$ and r_q ($-r_\mathrm{f}$ and r_f) with external coupling rate of $\gamma_\mathrm{ex}^\mathrm{q}/4$ ($\gamma_\mathrm{ex}^\mathrm{f}/4$), where it is assumed that $r_\mathrm{q} \leq r_\mathrm{f}$.

Cooperative effects mediated by a waveguide

We derive the cooperative effects on the qubit and the JQF mediated by the waveguide. Setting $\hbar = v = 1$, where v is the velocity of the microwaves, the total Hamiltonian under the rotating wave approximation is given by

$$\hat{H} = \hat{H}_\mathrm{sys} + \int_{-\infty}^{\infty} dk\, k\hat{a}_k^\dagger \hat{a}_k + \sum_{i=\mathrm{q,f}} \int_{-\infty}^{\infty} \frac{dk}{\sqrt{2\pi}} \sqrt{\frac{\gamma_\mathrm{ex}^i}{4}} \left(\hat{b}_i^\dagger \hat{a}_k \mathrm{e}^{-\mathrm{i}kr_i} + \hat{b}_i \hat{a}_k^\dagger \mathrm{e}^{+\mathrm{i}kr_i} + \hat{b}_i^\dagger \hat{a}_k \mathrm{e}^{+\mathrm{i}kr_i} + \hat{b}_i \hat{a}_k^\dagger \mathrm{e}^{-\mathrm{i}kr_i} \right), \tag{1}$$

where $\hat{H}_\mathrm{sys} = \sum_{i=\mathrm{q,f}} \omega_i \hat{b}_i^\dagger \hat{b}_i + \frac{\alpha_i}{2} \hat{b}_i^{\dagger 2} \hat{b}_i^2$ is the system Hamiltonian of the two transmon qubits with resonance frequencies ω_i and anharmonicities α_i, $\hat{b}_i$ is the annihilation operator of the transmon qubit i, and $\hat{a}_k$ is the annihilation operator of the right-propagating mode with wavenumber k. Note that the rotating wave approximation should be valid as long as the resonance frequencies of the qubit and JQF are much larger than their external coupling rates. The coupling strength of the propagating mode and the system is assumed not to depend on the wavenumber. Furthermore, it is assumed that the lower limit of k integration is extended to $-\infty$ since the propagating mode with a negative frequency does not affect the dynamics due to the large detuning from the system frequencies. The waveguide mode operator in the wavenumber representation, $\hat{a}_k$, is associated with that in the real-space representation, $\hat{a}_r$, as

$$\hat{a}_k = \int_{-\infty}^{\infty} \frac{dr}{\sqrt{2\pi}} \hat{a}_r \mathrm{e}^{-\mathrm{i}kr} \quad \text{and} \quad \hat{a}_r = \int_{-\infty}^{\infty} \frac{dk}{\sqrt{2\pi}} \hat{a}_k \mathrm{e}^{\mathrm{i}kr}. \tag{2}$$

They obey the following commutation relations: $\left[\hat{a}_k, \hat{a}_{k'}^\dagger\right] = \delta(k-k')$ and $\left[\hat{a}_r, \hat{a}_{r'}^\dagger\right] = \delta(r-r')$.

From Supplementary Equation 1, the Heisenberg equation for $\hat{a}_k$ is given by

$$\frac{d\hat{a}_k}{dt} = -\mathrm{i}k\hat{a}_k - \frac{\mathrm{i}}{\sqrt{2\pi}} \sum_{i=\mathrm{q,f}} \sqrt{\frac{\gamma_\mathrm{ex}^i}{4}} \left(\hat{b}_i \mathrm{e}^{+\mathrm{i}kr_i} + \hat{b}_i \mathrm{e}^{-\mathrm{i}kr_i} \right), \tag{3}$$

which can be formally solved as

$$\hat{a}_k = \hat{a}_k(0)\mathrm{e}^{-\mathrm{i}kt} - \frac{\mathrm{i}}{\sqrt{2\pi}} \sum_{i=\mathrm{q,f}} \sqrt{\frac{\gamma_\mathrm{ex}^i}{4}} \int_0^t dt' \left(\hat{b}_i(t')\mathrm{e}^{-\mathrm{i}k(-r_i - t' + t)} + \hat{b}_i(t')\mathrm{e}^{-\mathrm{i}k(r_i - t' + t)} \right), \tag{4}$$

where $\hat{a}_k(0)$ is the right-propagating annihilation operator with wavenumber k at the initial time $t = 0$ and $\hat{b}_i(t')$ is the annihilation operator of the transmon qubit at time t'. Note that operators at time t are written in a simple form hereinafter, e.g. $\hat{a}_k = \hat{a}_k(t)$. Multiplying Supplementary Equation 4 by $\mathrm{e}^{\mathrm{i}kr}/\sqrt{2\pi}$ and integrating from $-\infty$ to ∞ with respect to k, we obtain the input-output relation of the right-propagating mode in the real-space representation as

$$\hat{a}_r = \hat{a}_{r-t}(0) - \mathrm{i}\sum_{i=\mathrm{q,f}} \sqrt{\frac{\gamma^i_{\mathrm{ex}}}{4}} \left\{ \Theta_{r\in(-r_i,-r_i+t)}\, \hat{b}_i(t-r-r_i) + \Theta_{r\in(r_i,r_i+t)}\, \hat{b}_i(t-r+r_i) \right\}, \tag{5}$$

where Θ is a product of Heaviside step functions, $\Theta_{r\in(a,b)} = \theta(r-a)\theta(b-r)$.

Using Supplementary Equations 1 and 2, the time evolution of an arbitrary operator $\hat{O}$ supported by the two-qubit subspace is described in the Heisenberg picture as

$$\frac{d\hat{O}}{dt} = \mathrm{i}\left[\hat{H}_{\mathrm{sys}}, \hat{O}\right] + \mathrm{i}\sum_{i=\mathrm{q,f}} \sqrt{\frac{\gamma^i_{\mathrm{ex}}}{4}} \left(\left[\hat{b}^\dagger_i, \hat{O}\right] (\hat{a}_{-r_i} + \hat{a}_{r_i}) + (\hat{a}^\dagger_{-r_i} + \hat{a}^\dagger_{r_i}) \left[\hat{b}_i, \hat{O}\right] \right). \tag{6}$$

By substituting Supplementary Equation 5 into Supplementary Equation 6, it can be rewritten as

$$\begin{aligned}\frac{d\hat{O}}{dt} =& \,\mathrm{i}\left[\hat{H}_{\mathrm{sys}}, \hat{O}\right] + \mathrm{i}\sum_{i=\mathrm{q,f}} \sqrt{\gamma^i_{\mathrm{ex}}} \left(\left[\hat{b}^\dagger_i, \hat{O}\right] \frac{\hat{a}_{-r_i-t}(0) + \hat{a}_{r_i-t}(0)}{2} + \frac{\hat{a}^\dagger_{-r_i-t}(0) + \hat{a}^\dagger_{r_i-t}(0)}{2} \left[\hat{b}_i, \hat{O}\right] \right) \\ &+ \sum_{i=\mathrm{q,f}} \frac{\gamma^i_{\mathrm{ex}}}{2} \left(\left[\hat{b}^\dagger_i, \hat{O}\right] \frac{\hat{b}_i + \hat{b}_i(t-2r_i)}{2} - \frac{\hat{b}^\dagger_i + \hat{b}^\dagger_i(t-2r_i)}{2} \left[\hat{b}_i, \hat{O}\right] \right) \\ &+ \sum_{i=\mathrm{q,f}} \frac{\sqrt{\gamma^{\mathrm{q}}_{\mathrm{ex}}\gamma^{\mathrm{f}}_{\mathrm{ex}}}}{2} \left(\left[\hat{b}^\dagger_i, \hat{O}\right] \frac{\hat{b}_{\bar{i}}(t+r_{\mathrm{q}}-r_{\mathrm{f}}) + \hat{b}_{\bar{i}}(t-r_{\mathrm{q}}-r_{\mathrm{f}})}{2} - \frac{\hat{b}^\dagger_{\bar{i}}(t+r_{\mathrm{q}}-r_{\mathrm{f}}) + \hat{b}^\dagger_{\bar{i}}(t-r_{\mathrm{q}}-r_{\mathrm{f}})}{2} \left[\hat{b}_i, \hat{O}\right] \right),\end{aligned} \tag{7}$$

where $\bar{i} = \mathrm{f}$ ($\bar{i} = \mathrm{q}$) if $i = \mathrm{q}$ ($i = \mathrm{f}$).

Next, we employ the free-evolution approximation, $\hat{b}_i(t-x) \approx \hat{b}_i \mathrm{e}^{\mathrm{i}\omega_i x}$, which is valid when the time delay is much shorter than the typical time scales of dynamics of the qubits, such as the radiative lifetime. Furthermore, the initial state of the propagating mode is assumed to be separable from the system of the qubit and JQF and to be either in the vacuum state or a coherent state. Since a coherent state, which generally includes the vacuum state, is an eigenstate of the annihilation operator with its eigenvalue being a coherent amplitude, the annihilation operator of the propagating mode at the initial time $t = 0$ can be replaced with its complex amplitude, e.g. $\hat{a}_{-t}(0) \to \sqrt{\dot{n}}\, \mathrm{e}^{-\mathrm{i}\omega t}$, where $\dot{n}$ is the photon flux of the coherent drive field and ω is the drive frequency. Then, Supplementary Equation 7 is rewritten as

$$\begin{aligned}\frac{d\hat{O}}{dt} =& \,\mathrm{i}\left[\hat{H}_{\mathrm{sys}}, \hat{O}\right] + \mathrm{i}\sum_{i=\mathrm{q,f}} \sqrt{\gamma^i_{\mathrm{ex}}\dot{n}}\, \cos(\omega r_i) \left[\hat{b}^\dagger_i \mathrm{e}^{-\mathrm{i}\omega t} + \hat{b}_i \mathrm{e}^{\mathrm{i}\omega t}, \hat{O}\right] \\ &+ \sum_{i=\mathrm{q,f}} \frac{\gamma^i_{\mathrm{ex}} \cos(\omega_i r_i)}{2} \left(\left[\hat{b}^\dagger_i, \hat{O}\right] \hat{b}_i \mathrm{e}^{\mathrm{i}\omega_i r_i} - \hat{b}^\dagger_i \left[\hat{b}_i, \hat{O}\right] \mathrm{e}^{-\mathrm{i}\omega_i r_i} \right) \\ &+ \sum_{i=\mathrm{q,f}} \frac{\sqrt{\gamma^{\mathrm{q}}_{\mathrm{ex}}\gamma^{\mathrm{f}}_{\mathrm{ex}}}\, \cos(\omega_{\bar{i}} r_{\mathrm{q}})}{2} \left(\left[\hat{b}^\dagger_i, \hat{O}\right] \hat{b}_{\bar{i}} \mathrm{e}^{\mathrm{i}\omega_{\bar{i}} r_{\mathrm{f}}} - \hat{b}^\dagger_{\bar{i}} \mathrm{e}^{-\mathrm{i}\omega_{\bar{i}} r_{\mathrm{f}}} \left[\hat{b}_i, \hat{O}\right] \right).\end{aligned} \tag{8}$$

The second term on the right-hand side is associated with the coherent-drive Hamiltonian of the qubit and the JQF, which is defined by $\hat{H}_{\mathrm{drive}} = \sum_{i=\mathrm{q,f}} \sqrt{\gamma^i_{\mathrm{ex}}\dot{n}}\, \cos(\omega r_i) \left(\hat{b}^\dagger_i \mathrm{e}^{-\mathrm{i}\omega t} + \hat{b}_i \mathrm{e}^{\mathrm{i}\omega t}\right)$.

Using the cyclic invariance of trace operations, the time derivative of the expectation value $\langle\hat{O}\rangle$ is described in the

Schrödinger picture as

$$\frac{d\langle\hat{O}\rangle}{dt} = \mathrm{Tr}\left[\frac{d\hat{O}}{dt}\,\hat{\rho}_{\mathrm{T}}(0)\right] = \mathrm{Tr}\left[\hat{O}(0)\,\frac{d\hat{\rho}_{\mathrm{T}}}{dt}\right]$$
$$= -\,\mathrm{i}\;\mathrm{Tr}\left[\hat{O}\,[\hat{H}_{\mathrm{sys}} + \hat{H}_{\mathrm{drive}},\, \hat{\rho}_{\mathrm{T}}]\right]$$
$$+ \sum_{i=\mathrm{q,f}} \mathrm{Tr}\left[\hat{O}\frac{\gamma_{\mathrm{ex}}^{i}\cos(\omega_i r_i)}{2}\left\{\left(\hat{b}_i\hat{\rho}_{\mathrm{T}}\hat{b}_i^{\dagger} - \hat{b}_i^{\dagger}\hat{b}_i\hat{\rho}_{\mathrm{T}}\right)\mathrm{e}^{\mathrm{i}\omega_i r_i} + \left(\hat{b}_i\hat{\rho}_{\mathrm{T}}\hat{b}_i^{\dagger} - \hat{\rho}_{\mathrm{T}}\hat{b}_i^{\dagger}\hat{b}_i\right)\mathrm{e}^{-\mathrm{i}\omega_i r_i}\right\}\right] \tag{9}$$
$$+ \sum_{i=\mathrm{q,f}} \mathrm{Tr}\left[\hat{O}\,\frac{\sqrt{\gamma_{\mathrm{ex}}^{\mathrm{q}}\gamma_{\mathrm{ex}}^{\mathrm{f}}}\,\cos(\omega_{\bar{i}} r_q)}{2}\left\{\left(\hat{b}_{\bar{i}}\hat{\rho}_{\mathrm{T}}\hat{b}_i^{\dagger} - \hat{b}_i^{\dagger}\hat{b}_{\bar{i}}\hat{\rho}_{\mathrm{T}}\right)\mathrm{e}^{\mathrm{i}\omega_{\bar{i}} r_{\mathrm{f}}} + \left(\hat{b}_i\hat{\rho}_{\mathrm{T}}\hat{b}_{\bar{i}}^{\dagger} - \hat{\rho}_{\mathrm{T}}\hat{b}_{\bar{i}}^{\dagger}\hat{b}_i\right)\mathrm{e}^{-\mathrm{i}\omega_{\bar{i}} r_{\mathrm{f}}}\right\}\right].$$

The expectation value of the local operator $\hat{O}$ can be obtained from the reduced density matrix, in which the degrees of freedom of the propagating modes are traced out. Therefore, by defining the density-matrix operator of the composite system of the qubit and JQF as $\rho = \mathrm{Tr}_{\mathrm{p}}[\rho_{\mathrm{T}}]$, where $\mathrm{Tr}_{\mathrm{p}}[\cdot]$ is the partial trace of the propagating mode, the reduced master equation is derived from Supplementary Equation 9 as

$$\frac{d\hat{\rho}}{dt} = -\,\mathrm{i}\left[\hat{H}_{\mathrm{sys}} + \hat{H}_{\mathrm{drive}},\, \hat{\rho}\right]$$
$$+ \sum_{i=\mathrm{q,f}} \frac{\gamma_{\mathrm{ex}}^{i}\cos(\omega_i r_i)}{2}\left[\left(\hat{b}_i\hat{\rho}\hat{b}_i^{\dagger} - \hat{b}_i^{\dagger}\hat{b}_i\hat{\rho}\right)\mathrm{e}^{\mathrm{i}\omega_i r_i} + \left(\hat{b}_i\hat{\rho}\hat{b}_i^{\dagger} - \hat{\rho}\hat{b}_i^{\dagger}\hat{b}_i\right)\mathrm{e}^{-\mathrm{i}\omega_i r_i}\right] \tag{10}$$
$$+ \sum_{i=\mathrm{q,f}} \frac{\sqrt{\gamma_{\mathrm{ex}}^{\mathrm{q}}\gamma_{\mathrm{ex}}^{\mathrm{f}}}\,\cos(\omega_{\bar{i}} r_q)}{2}\left[\left(\hat{b}_{\bar{i}}\hat{\rho}\hat{b}_i^{\dagger} - \hat{b}_i^{\dagger}\hat{b}_{\bar{i}}\hat{\rho}\right)\mathrm{e}^{\mathrm{i}\omega_{\bar{i}} r_{\mathrm{f}}} + \left(\hat{b}_i\hat{\rho}\hat{b}_{\bar{i}}^{\dagger} - \hat{\rho}\hat{b}_{\bar{i}}^{\dagger}\hat{b}_i\right)\mathrm{e}^{-\mathrm{i}\omega_{\bar{i}} r_{\mathrm{f}}}\right].$$

Then, the master equation can be rewritten in the Lindblad form as

$$\frac{d\hat{\rho}}{dt} = -\,\mathrm{i}\left[\hat{H}_{\mathrm{sys}} + \hat{H}_{\mathrm{drive}} + \hat{H}_{\mathrm{Lamb}} + \hat{H}_{\mathrm{eff}},\, \hat{\rho}\right] + \sum_{i,j=\mathrm{q,f}} \gamma_{\mathrm{ex}}^{ij}\,\mathcal{D}(\hat{b}_i, \hat{b}_j)\hat{\rho}, \tag{11}$$

where the Lamb-shift Hamiltonian is given by $\hat{H}_{\mathrm{Lamb}} = \sum_{i=\mathrm{q,f}} \frac{\gamma_{\mathrm{ex}}^{i}}{4}\sin(2\omega_i r_i)\,\hat{b}_i^{\dagger}\hat{b}_i$. The waveguide-mediated effective interaction Hamiltonian is given by

$$\hat{H}_{\mathrm{eff}} = J\,\hat{b}_{\mathrm{q}}^{\dagger}\hat{b}_{\mathrm{f}} + J^{*}\,\hat{b}_{\mathrm{f}}^{\dagger}\hat{b}_{\mathrm{q}}, \tag{12}$$

where $J = \sqrt{\gamma_{\mathrm{ex}}^{\mathrm{q}}\gamma_{\mathrm{ex}}^{\mathrm{f}}}/2\,[\cos(\omega_{\mathrm{f}} r_{\mathrm{q}})\mathrm{e}^{\mathrm{i}\omega_{\mathrm{f}} r_{\mathrm{f}}} - \cos(\omega_{\mathrm{q}} r_{\mathrm{q}})\mathrm{e}^{-\mathrm{i}\omega_{\mathrm{q}} r_{\mathrm{f}}}]/(2\mathrm{i})$ is the coupling strength. Furthermore, we define the individual and correlated radiative decay rates of the qubit and the JQF as $\gamma_{\mathrm{ex}}^{ii} = \gamma_{\mathrm{ex}}^{i}\cos^2(\omega_i r_i)$ and $\gamma_{\mathrm{ex}}^{\mathrm{qf}} = \gamma_{\mathrm{ex}}^{\mathrm{fq}*} = \sqrt{\gamma_{\mathrm{ex}}^{\mathrm{q}}\gamma_{\mathrm{ex}}^{\mathrm{f}}}\,[\cos(\omega_{\mathrm{f}} r_{\mathrm{q}})\mathrm{e}^{\mathrm{i}\omega_{\mathrm{f}} r_{\mathrm{f}}} + \cos(\omega_{\mathrm{q}} r_{\mathrm{q}})\mathrm{e}^{-\mathrm{i}\omega_{\mathrm{q}} r_{\mathrm{f}}}]/2$, respectively. The superoperator for the decay terms is defined as $\mathcal{D}(\hat{A}, \hat{B})\hat{\rho} = \hat{B}\hat{\rho}\hat{A}^{\dagger} - (\hat{A}^{\dagger}\hat{B}\hat{\rho} + \hat{\rho}\hat{A}^{\dagger}\hat{B})/2$.

Without loss of generality, the resonance frequencies of the qubit and JQF can be renormalized such that the Lamb shifts are included in the system Hamiltonian, i.e. $\hat{H}_{\mathrm{Lamb}} = 0$. In our device, the qubit is located at the open end of the semi-infinite waveguide ($r_{\mathrm{q}} = 0$) and the JQF is placed at the distance d apart from the qubit ($r_{\mathrm{f}} = d$). Furthermore, the imperfections of the qubit and JQF, such as the intrinsic relaxation and the pure dephasing, are added to simulate the real device. Note that we assume that the control line is cooled down enough as we have checked it in the almost same configuration [3]. As a result, the master equation of the composite system containing the qubit and JQF is described as

$$\frac{d\hat{\rho}}{dt} = -\,\mathrm{i}\left[\hat{H}_{\mathrm{sys}} + \hat{H}_{\mathrm{drive}} + \hat{H}_{\mathrm{eff}},\, \hat{\rho}\right] + \sum_{i,j=\mathrm{q,f}} \gamma_{\mathrm{ex}}^{ij}\,\mathcal{D}(\hat{b}_i, \hat{b}_j)\hat{\rho}$$
$$+ \sum_{i=\mathrm{q,f}}\left[\gamma_{\mathrm{in}}^{i}(1 + n_{\mathrm{th}}^{i})\,\mathcal{D}(\hat{b}_i)\hat{\rho} + \gamma_{\mathrm{in}}^{i}n_{\mathrm{th}}^{i}\,\mathcal{D}(\hat{b}_i^{\dagger})\hat{\rho} + \gamma_{\phi}^{i}\,\mathcal{D}(\hat{b}_i^{\dagger}\hat{b}_i)\hat{\rho}\right], \tag{13}$$

where γ_{in}^{i}, γ_{ϕ}^{i} and n_{th}^{i} are the intrinsic relaxation rate, pure dephasing rate, and thermal quanta of the intrinsic loss channel, respectively. The individual dissipation terms, which appear in the second line of Supplementary Equation 13,

are simplified as $\mathcal{D}(\hat{A}) = \mathcal{D}(\hat{A}, \hat{A})$. Here, the system Hamiltonian in the frame rotating at drive frequency ω is given by

$$\hat{H}_{\text{sys}} = \sum_{i=\text{q,f}} (\omega_i - \omega)\, \hat{b}_i^\dagger \hat{b}_i + \frac{\alpha_i}{2} \hat{b}_i^{\dagger 2} \hat{b}_i^2. \tag{14}$$

The drive Hamiltonian in the rotating frame is given by

$$\hat{H}_{\text{drive}} = \sqrt{\gamma_{\text{ex}}^{\text{q}} \dot{n}} \left(\hat{b}_{\text{q}}^\dagger + \hat{b}_{\text{q}}\right) + \sqrt{\gamma_{\text{ex}}^{\text{f}} \dot{n}}\, \cos(\omega d) \left(\hat{b}_{\text{f}}^\dagger + \hat{b}_{\text{f}}\right). \tag{15}$$

The waveguide-mediated coupling strength is given by

$$J = \frac{\sqrt{\gamma_{\text{ex}}^{\text{q}} \gamma_{\text{ex}}^{\text{f}}}}{2} \frac{\mathrm{e}^{\mathrm{i}\omega_{\text{f}} d} - \mathrm{e}^{-\mathrm{i}\omega_{\text{q}} d}}{2i}. \tag{16}$$

The individual and correlated radiative decay rates to the waveguide are given by

$$\gamma_{\text{ex}}^{\text{qq}} = \gamma_{\text{ex}}^{\text{q}}, \quad \gamma_{\text{ex}}^{\text{ff}} = \gamma_{\text{ex}}^{\text{f}} \cos^2(\omega_{\text{f}} d) \tag{17}$$

and

$$\gamma_{\text{ex}}^{\text{qf}} = \gamma_{\text{ex}}^{\text{fq}*} = \sqrt{\gamma_{\text{ex}}^{\text{q}} \gamma_{\text{ex}}^{\text{f}}}\, \frac{\mathrm{e}^{\mathrm{i}\omega_{\text{f}} d} + \mathrm{e}^{-\mathrm{i}\omega_{\text{q}} d}}{2}, \tag{18}$$

respectively. We calculate the numerical results of the dynamics of the qubit with the JQF using this master equation with the parameters listed in Supplementary Table I.

Bright and dark modes

The individual and correlated decays of the qubit and JQF can be understood as the individual decays of the bright and dark modes [4], which are defined by

$$\hat{b}_{\text{B/D}} = \frac{\left(\gamma_{\text{ex}}^{\text{B/D}} - \gamma_{\text{ex}}^{\text{ff}}\right) \hat{b}_{\text{q}} + \gamma_{\text{ex}}^{\text{fq}*}\, \hat{b}_{\text{f}}}{\sqrt{\left(\gamma_{\text{ex}}^{\text{B/D}} - \gamma_{\text{ex}}^{\text{ff}}\right)^2 + \left|\gamma_{\text{ex}}^{\text{qf}}\right|^2}} \tag{19}$$

with decay rates

$$\gamma_{\text{ex}}^{\text{B/D}} = \frac{\gamma_{\text{ex}}^{\text{qq}} + \gamma_{\text{ex}}^{\text{ff}}}{2} \pm \sqrt{\left(\frac{\gamma_{\text{ex}}^{\text{qq}} - \gamma_{\text{ex}}^{\text{ff}}}{2}\right)^2 + \left|\gamma_{\text{ex}}^{\text{qf}}\right|^2}. \tag{20}$$

Therefore, the radiative decay terms in Supplementary Equation 13 can be replaced by

$$\sum_{i,j=\text{q,f}} \gamma_{\text{ex}}^{ij}\, \mathcal{D}(\hat{b}_i, \hat{b}_j)\hat{\rho} \;\rightarrow \sum_{\mu=\text{B,D}} \gamma_{\text{ex}}^{\mu}\, \mathcal{D}(\hat{b}_\mu)\hat{\rho}. \tag{21}$$

Reflection spectrum of a single qubit

As discussed in Supplementary Notes 5 and 7, the qubit and JQF are first characterized individually by measuring their reflection spectra via the control line. Therefore, we here provide a model of one of the transmon qubits (the qubit or the JQF) coupled to the semi-infinite waveguide.

By setting one of the external coupling rates of the qubit and JQF to zero in the previous model, the master equation of the single transmon qubit i is obtained as

$$\frac{d\hat{\rho}}{dt} = -\mathrm{i}\left[\hat{H}_{\text{sys}} + \hat{H}_{\text{drive}},\, \hat{\rho}\right] + \gamma_{\text{ex}}^{i} \cos^2(\omega_i r_i)\, \mathcal{D}(\hat{b}_i)\hat{\rho} + \gamma_{\text{in}}(1 + n_{\text{th}}^{i})\, \mathcal{D}(\hat{b}_i)\hat{\rho} + \gamma_{\text{in}}^{i} n_{\text{th}}^{i}\, \mathcal{D}(\hat{b}_i^\dagger)\hat{\rho} + \gamma_{\phi}^{i}\, \mathcal{D}(\hat{b}_i^\dagger \hat{b}_i)\hat{\rho}, \tag{22}$$

where ρ is the density-matrix operator of the transmon qubit, $\hat{H}_{\text{sys}} = (\omega_i - \omega)\,\hat{b}_i^\dagger \hat{b}_i + \frac{\alpha_i}{2}\hat{b}_i^{\dagger 2}\hat{b}_i^2$ and $\hat{H}_{\text{drive}} = \sqrt{\gamma_{\text{ex}}^i \dot{n}}\cos(\omega_i r_i)\left(\hat{b}_i^\dagger + \hat{b}_i\right)$. Note that the drive amplitude is assumed to be constant with respect to the drive frequency, i.e. $\cos(\omega r_i) \approx \cos(\omega_i r_i)$, since the drive frequency is close to that of the transmon qubit.

The reflection coefficient of the transmon qubit is numerically obtained by using the steady-state solution and the input-output relation. The steady state $\hat{\rho}_{\text{ss}}$ of the transmon qubit in the frame rotating at the drive frequency can be obtained by solving Supplementary Equation 22 with $d\hat{\rho}/dt \to 0$. Then, the expectation value of the annihilation operator of the transmon qubit in the steady state is obtained at time t in the laboratory frame as

$$\langle \hat{b}_i(t)\rangle = \text{Tr}\left[\left(\hat{b}_i \mathrm{e}^{-\mathrm{i}\omega t}\right)\hat{\rho}_{\text{ss}}\right] = \langle \hat{b}_i\rangle_{\text{ss}}\mathrm{e}^{-\mathrm{i}\omega t}, \tag{23}$$

where $\langle \hat{b}_i\rangle_{\text{ss}} = \text{Tr}\left[\hat{b}_i\hat{\rho}_{\text{ss}}\right]$. When only a single qubit is coupled to the control line, the input-output relation of Supplementary Equation 5 is simplified as

$$\hat{a}_r = \hat{a}_{r-t}(0) - \mathrm{i}\sqrt{\gamma_{\text{ex}}^i}\cos(\omega_i r_i)\,\hat{b}_i(t-r), \tag{24}$$

where the position r should be on the right-hand side of all coupling positions in the model shown in Supplementary Figure 2b. Using Supplementary Equation 23, the complex amplitude of the output field in the steady state can be represented as

$$\begin{aligned}\langle \hat{a}_r\rangle &= \langle \hat{a}_{r-t}(0)\rangle - \mathrm{i}\sqrt{\gamma_{\text{ex}}^i}\cos(\omega_i r_i)\,\langle \hat{b}_i(t-r)\rangle \\ &= \langle \hat{a}_{r-t}(0)\rangle - \mathrm{i}\sqrt{\gamma_{\text{ex}}^i}\cos(\omega_i r_i)\,\langle \hat{b}_i\rangle_{\text{ss}}\,\mathrm{e}^{-\mathrm{i}\omega(t-r)}.\end{aligned} \tag{25}$$

The reflection spectrum is then obtained as

$$\mathrm{S}_{11}(\omega) = \frac{\langle \hat{a}_r\rangle}{\langle \hat{a}_{r-t}(0)\rangle} = 1 - \mathrm{i}\sqrt{\frac{\gamma_{\text{ex}}^i}{\dot{n}}}\cos(\omega_i r_i)\,\langle \hat{b}_i\rangle_{\text{ss}}, \tag{26}$$

where $\langle a_{r-t}(0)\rangle = \sqrt{\dot{n}}\,\mathrm{e}^{-\mathrm{i}\omega(t-r)}$ according to our earlier assumption.

Supplementary Note 4: Working principle of JQF

We now explain the working principle of the JQF: why the JQF suppresses the qubit radiative decay when a drive is switched off and why the JQF does not reduce the qubit Rabi frequency under a strong drive. For simplicity, we consider a case where two-level systems are used for the qubit and JQF. In other words, we replace the annihilation operator $\hat{b}_i$ with the lowering operator of the Pauli matrix $\hat{\sigma}_i$ in the model discussed in Supplementary Note 3. After that, we consider a transmon JQF and study the dependence of the JQF anharmonicity on the Rabi frequency and Rabi decay time of the qubit.

Requirements for JQF

As we discuss in the main text, we have three requirements for the JQF to act as a filter for the qubit:

- The JQF and qubit should be on resonance ($\omega_{\text{f}} = \omega_{\text{q}}$),
- The distance between the qubit and JQF should be half the qubit wavelength ($d = \lambda_{\text{q}}/2$), and
- The JQF should be coupled to the control line much more strongly than the qubit ($\gamma_{\text{ex}}^{\text{f}} \gg \gamma_{\text{ex}}^{\text{q}}$).

The first condition ($\omega_{\text{f}} = \omega_{\text{q}}$) is required to maximize the cooperative effects between the qubit and JQF. The strength of the exchange interaction and the correlated decay rate are given by $J = \frac{\sqrt{\gamma_{\text{ex}}^{\text{q}}\gamma_{\text{ex}}^{\text{f}}}}{2}\sin(\omega_{\text{q}} d)$ and $\gamma_{\text{ex}}^{\text{qf}} = \gamma_{\text{ex}}^{\text{fq}} = \sqrt{\gamma_{\text{ex}}^{\text{q}}\gamma_{\text{ex}}^{\text{f}}}\cos(\omega_{\text{q}} d)$, respectively. The second condition ($d = \lambda_{\text{q}}/2$) is needed to suppress the exchange interaction, i.e. $J = 0$, to prevent the qubit from hybridizing with the JQF, and to maximize the correlated decay, i.e. $\gamma_{\text{ex}}^{\text{qf}} = \gamma_{\text{ex}}^{\text{fq}} = -\sqrt{\gamma_{\text{ex}}^{\text{q}}\gamma_{\text{ex}}^{\text{f}}}$. Using Supplementary Equations 19 and 20, the bright and dark modes are given by

$$\hat{\sigma}_{\text{B}} = \frac{1}{\sqrt{\gamma_{\text{ex}}^{\text{q}} + \gamma_{\text{ex}}^{\text{f}}}}\left(-\sqrt{\gamma_{\text{ex}}^{\text{q}}}\,\hat{\sigma}_{\text{q}} + \sqrt{\gamma_{\text{ex}}^{\text{f}}}\,\hat{\sigma}_{\text{f}}\right) \tag{27}$$

and

$$\hat{\sigma}_{\mathrm{D}} = \frac{1}{\sqrt{\gamma_{\mathrm{ex}}^{\mathrm{q}} + \gamma_{\mathrm{ex}}^{\mathrm{f}}}} \left(\sqrt{\gamma_{\mathrm{ex}}^{\mathrm{f}}}\, \hat{\sigma}_{\mathrm{q}} + \sqrt{\gamma_{\mathrm{ex}}^{\mathrm{q}}}\, \hat{\sigma}_{\mathrm{f}} \right) \tag{28}$$

with decay rates of $\gamma_{\mathrm{ex}}^{\mathrm{B}} = \gamma_{\mathrm{ex}}^{\mathrm{q}} + \gamma_{\mathrm{ex}}^{\mathrm{f}}$ and $\gamma_{\mathrm{ex}}^{\mathrm{D}} = 0$. The third condition ($\gamma_{\mathrm{ex}}^{\mathrm{f}} \gg \gamma_{\mathrm{ex}}^{\mathrm{q}}$) causes the dark mode to be close to the qubit mode, i.e. $\hat{\sigma}_{\mathrm{D}} \approx \hat{\sigma}_{\mathrm{q}}$, suppressing the radiative decay of the qubit. On the other hand, the bright mode becomes close to the JQF mode, i.e. $\hat{\sigma}_{\mathrm{B}} \approx \hat{\sigma}_{\mathrm{f}}$.

Dynamics of qubit with JQF

Using the bright-mode basis, the master equation (Supplementary Equation 13) with the resonant control field ($\omega = \omega_{\mathrm{q}}$) can be rewritten as

$$\frac{d\hat{\rho}}{dt} = -\mathrm{i} \left[\hat{H}_{\mathrm{drive}},\, \hat{\rho} \right] + \gamma_{\mathrm{ex}}^{\mathrm{B}}\, \mathcal{D}(\hat{\sigma}_{\mathrm{B}})\hat{\rho}, \tag{29}$$

where $\hat{H}_{\mathrm{drive}} = -\sqrt{\gamma_{\mathrm{ex}}^{B}\dot{n}}\ (\hat{\sigma}_{\mathrm{B}}^{\dagger} + \hat{\sigma}_{\mathrm{B}})$. For simplicity, we neglect the imperfections of the qubit and the JQF as $\gamma_{\mathrm{in}}^{i} = \gamma_{\phi}^{i} = 0$.

Here, we describe the master equation with the bright- and dark modes basis: the ground state $|\mathrm{gg}\rangle$, the excited state of the bright mode $|\widetilde{\mathrm{ge}}\rangle = \hat{\sigma}_{\mathrm{B}}^{\dagger}|\mathrm{gg}\rangle$, the excited state of the dark mode $|\widetilde{\mathrm{eg}}\rangle = \hat{\sigma}_{\mathrm{D}}^{\dagger}|\mathrm{gg}\rangle$, and both excited states $|\mathrm{ee}\rangle$. In this representation, $|\mathrm{gg}\rangle$, $|\mathrm{ge}\rangle$, $|\mathrm{eg}\rangle$, and $|\mathrm{ee}\rangle$ are the product states of the ground and excited states of the qubit and the two-level JQF. Using the bright- and dark-modes basis, the lowering operator of the bright mode multiplied by the coupling coefficient $\sqrt{\gamma_{\mathrm{ex}}^{\mathrm{B}}}$, which corresponds to the transition moment of the bright mode by the control field, can be rewritten exactly as

$$\sqrt{\gamma_{\mathrm{ex}}^{\mathrm{B}}}\, \hat{\sigma}_{\mathrm{B}} = \sqrt{\gamma_{\mathrm{ex}}^{\mathrm{B}}}\, |\mathrm{gg}\rangle\langle\widetilde{\mathrm{ge}}| + \frac{\gamma_{\mathrm{ex}}^{\mathrm{f}} - \gamma_{\mathrm{ex}}^{\mathrm{q}}}{\sqrt{\gamma_{\mathrm{ex}}^{\mathrm{B}}}}\, |\widetilde{\mathrm{eg}}\rangle\langle\mathrm{ee}| - 2\sqrt{\frac{\gamma_{\mathrm{ex}}^{\mathrm{q}}\gamma_{\mathrm{ex}}^{\mathrm{f}}}{\gamma_{\mathrm{ex}}^{\mathrm{B}}}}\, |\widetilde{\mathrm{ge}}\rangle\langle\mathrm{ee}|. \tag{30}$$

In the limit of $\gamma_{\mathrm{ex}}^{\mathrm{f}} \gg \gamma_{\mathrm{ex}}^{\mathrm{q}}$, it can be approximated as

$$\begin{aligned} \sqrt{\gamma_{\mathrm{ex}}^{\mathrm{B}}}\, \hat{\sigma}_{\mathrm{B}} &\approx \sqrt{\gamma_{\mathrm{ex}}^{\mathrm{f}}}\, |\mathrm{gg}\rangle\langle\mathrm{ge}| + \sqrt{\gamma_{\mathrm{ex}}^{\mathrm{f}}}\, |\mathrm{eg}\rangle\langle\mathrm{ee}| - 2\sqrt{\gamma_{\mathrm{ex}}^{\mathrm{q}}}\, |\mathrm{ge}\rangle\langle\mathrm{ee}| \\ &= \sqrt{\gamma_{\mathrm{ex}}^{\mathrm{f}}}\, \hat{\sigma}_{\mathrm{f}} - \sqrt{\gamma_{\mathrm{ex}}^{\mathrm{q}}}\, (1 + \hat{\sigma}_{z}^{\mathrm{f}})\hat{\sigma}_{\mathrm{q}}. \end{aligned} \tag{31}$$

This expression implies that the transition moment of the qubit depends on the state of the JQF, i.e. the qubit can be driven or decay only when the two-level JQF is in its excited state (see Supplementary Figure 3a). Using this approximation, the master equation (Supplementary Equation 29) can be rewritten as

$$\frac{d\hat{\rho}}{dt} = -\mathrm{i} \left[\hat{H}_{\mathrm{drive}},\, \hat{\rho} \right] + \mathcal{D} \left(\sqrt{\gamma_{\mathrm{ex}}^{\mathrm{f}}}\, \hat{\sigma}_{\mathrm{f}} - \sqrt{\gamma_{\mathrm{ex}}^{\mathrm{q}}}\, (1 + \hat{\sigma}_{z}^{\mathrm{f}})\hat{\sigma}_{\mathrm{q}} \right) \hat{\rho}, \tag{32}$$

where

$$\hat{H}_{\mathrm{drive}} = -\frac{\Omega_{\mathrm{f}}}{2}\hat{\sigma}_{x}^{\mathrm{f}} + \frac{\Omega_{\mathrm{q}}}{2}(1 + \hat{\sigma}_{z}^{\mathrm{f}})\hat{\sigma}_{x}^{\mathrm{q}} \tag{33}$$

is the approximative drive Hamiltonian where $\hat{\sigma}_{x}^{\mathrm{f}}$ and $\hat{\sigma}_{x}^{\mathrm{q}}$ are the Pauli x-matrix of the JQF and qubit, respectively. The Rabi frequencies are defined as $\Omega_{\mathrm{f}} = 2\sqrt{\gamma_{\mathrm{ex}}^{\mathrm{f}}\dot{n}}$ and $\Omega_{\mathrm{q}} = 2\sqrt{\gamma_{\mathrm{ex}}^{\mathrm{q}}\dot{n}}$.

As shown in Supplementary Figures 4a and b, we numerically calculate the Rabi frequency and Rabi decay time of the qubit as a function of the control amplitude using the rigorous model (blue solid line) of Supplementary Equation 29 and the approximative model (orange dashed line) of Supplementary Equation 32. The population of the ground state of the JQF is also calculated and is shown in Supplementary Figure 4c. The approximative model reproduces the results calculated with the rigorous model well. When the control field is large enough to saturate the JQF (see Supplementary Figure 4c), the Rabi frequency with the two-level JQF approaches that without the JQF (see Supplementary Figure 4a). On the other hand, the Rabi decay time of the qubit with the JQF is shorter than that without the JQF, even when the control field completely saturates the JQF (see Supplementary Figure 4b).

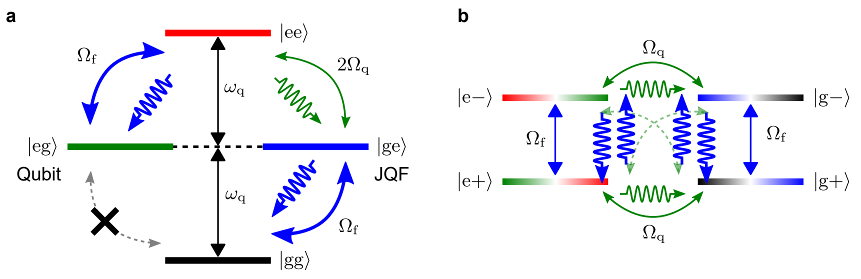

Supplementary Figure 3: Schematic of energy levels. **a**, Energy levels of the qubit and JQF in the eigenbasis of the system Hamiltonian in the laboratory frame. The blue and green solid arrows depict the coherent interactions, while the blue and green wavy arrows show the relaxations. The black arrows depict the qubit frequency in the laboratory frame. **b**, Energy levels of the qubit and JQF in the basis which diagonalizes the drive Hamiltonian of the JQF in the frame rotating at drive frequency $\omega = \omega_\mathrm{q}$. The blue solid arrows depict the frequency splittings due to the Rabi frequency of the JQF in the frame rotating at the drive frequency. The green solid and dashed arrows show the resonant and off-resonant coherent interactions of the qubit, respectively. The blue wavy arrows depict the dephasings in the Rabi oscillation of the JQF, while the green wavy arrows show the relaxations of the qubit.

Using the approximative master equation (32), we explain the working principle of the two-level JQF. When the JQF and qubit are driven by a control field, the drive Hamiltonian is further approximated as $\hat{H}_\mathrm{drive} \approx -\frac{\Omega_\mathrm{f}}{2}\hat{\sigma}_x^\mathrm{f} + \frac{\Omega_\mathrm{q}}{2}\hat{\sigma}_x^\mathrm{q}$, since the drive term $\frac{\Omega_\mathrm{f}}{2}\hat{\sigma}_x^\mathrm{f}$ suppresses the non-commutating coupling term $\frac{\Omega_\mathrm{q}}{2}\hat{\sigma}_z^\mathrm{f}\hat{\sigma}_x^\mathrm{q}$. This is known as the secular approximation. When the control field is switched off, the qubit decay immediately vanishes, since the JQF quickly decays to the ground state, resulting in $(1+\hat{\sigma}_z^\mathrm{f}) = 0$ in Supplementary Equation 32.

To understand the additional decay of the qubit Rabi oscillation caused by the two-level JQF as seen in Supplementary Figure 4b, we have to consider the perturbation of the coupling term $\frac{\Omega_\mathrm{q}}{2}\hat{\sigma}_z^\mathrm{f}\hat{\sigma}_x^\mathrm{q}$ suppressed by the secular approximation. We explain it by using the matrix representation.

From Supplementary Equation 33, the approximative drive Hamiltonian in the basis spanned by $\{|\mathrm{gg}\rangle, |\mathrm{ge}\rangle, |\mathrm{eg}\rangle, |\mathrm{ee}\rangle\}$ is given by

$$\hat{H}_\mathrm{drive} = \begin{pmatrix} 0 & -\frac{\Omega_\mathrm{f}}{2} & 0 & 0 \\ -\frac{\Omega_\mathrm{f}}{2} & 0 & 0 & \Omega_\mathrm{q} \\ 0 & 0 & 0 & -\frac{\Omega_\mathrm{f}}{2} \\ 0 & \Omega_\mathrm{q} & -\frac{\Omega_\mathrm{f}}{2} & 0 \end{pmatrix}. \tag{34}$$

By diagonalizing the drive terms of the JQF, the Hamiltonian is represented in the basis spanned by $\{|\mathrm{g}+\rangle, |\mathrm{g}-\rangle, |\mathrm{e}+\rangle, |\mathrm{e}-\rangle\}$ as

$$\hat{H}_\mathrm{drive} = \begin{pmatrix} -\frac{\Omega_\mathrm{f}}{2} & 0 & \frac{\Omega_\mathrm{q}}{2} & \cancel{-\frac{\Omega_\mathrm{q}}{2}} \\ 0 & \frac{\Omega_\mathrm{f}}{2} & \cancel{-\frac{\Omega_\mathrm{q}}{2}} & \frac{\Omega_\mathrm{q}}{2} \\ \frac{\Omega_\mathrm{q}}{2} & \cancel{-\frac{\Omega_\mathrm{q}}{2}} & -\frac{\Omega_\mathrm{f}}{2} & 0 \\ \cancel{-\frac{\Omega_\mathrm{q}}{2}} & \frac{\Omega_\mathrm{q}}{2} & 0 & \frac{\Omega_\mathrm{f}}{2} \end{pmatrix}, \tag{35}$$

where $|s\pm\rangle = \frac{1}{\sqrt{2}}(|s\mathrm{g}\rangle \pm |s\mathrm{e}\rangle)$ for $s = \mathrm{g}, \mathrm{e}$. We can neglect the off-resonant coupling elements (crossed out in Supplementary Equation 35) in the subspaces of $|\mathrm{g}+\rangle$ and $|\mathrm{e}-\rangle$ and of $|\mathrm{g}-\rangle$ and $|\mathrm{e}+\rangle$ since the detuning Ω_f is much larger than the coupling strength Ω_q, i.e. $\gamma_\mathrm{ex}^\mathrm{f} \gg \gamma_\mathrm{ex}^\mathrm{q}$, which corresponds to the secular approximation as explained above. As schematically shown with the green solid arrows in Supplementary Figure 3b, the qubit Rabi oscillations between $|\mathrm{g}+\rangle$ and $|\mathrm{e}+\rangle$ and between $|\mathrm{g}-\rangle$ and $|\mathrm{e}-\rangle$ occur with the expected Rabi frequency Ω_q.

As for the reduction of the Rabi decay time of the qubit, the additional decay channel can be explained by the same off-resonant coupling terms which we neglected using the secular approximation in the above explanation for the qubit Rabi oscillations. As schematically shown in Supplementary Figure 3b, the perturbation of the off-resonant coupling allows the qubit state to decay through the lossy JQF state (see the green dashed arrows and the blue wavy arrows). For example, consider the decay channel of the Rabi oscillation between $|\mathrm{g}+\rangle$ and $|\mathrm{e}+\rangle$. First, the decay from $|\mathrm{e}+\rangle$ to $|\mathrm{g}+\rangle$ with the rate of $\gamma_\mathrm{ex}^\mathrm{q}$ corresponds to the conventional radiative decay of the qubit to the control line. In addition, $|\mathrm{e}+\rangle$ ($|\mathrm{g}+\rangle$) decays to $|\mathrm{g}+\rangle$ ($|\mathrm{e}+\rangle$) via $|\mathrm{g}-\rangle$ ($|\mathrm{e}-\rangle$). Here, the mixing ratio of $|\mathrm{g}-\rangle$ to $|\mathrm{e}+\rangle$ ($|\mathrm{e}-\rangle$ to

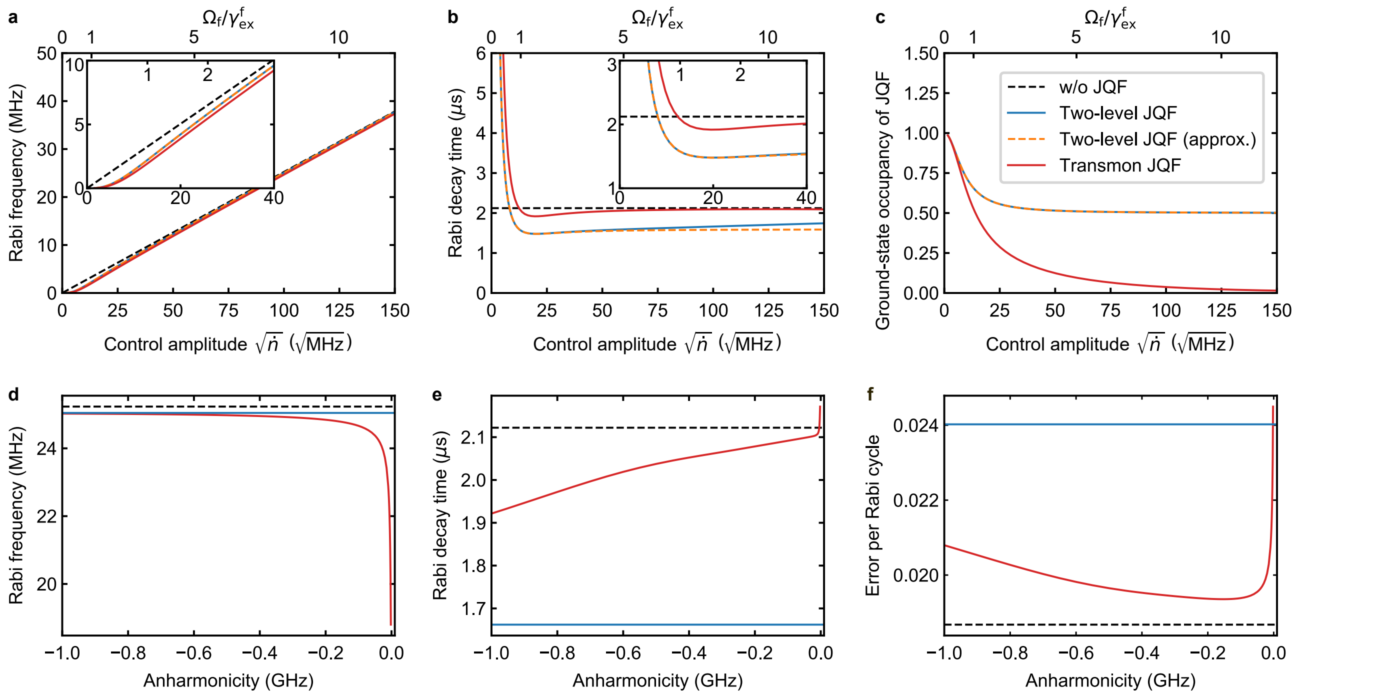

Supplementary Figure 4: Numerically simulated results of qubit control in the presence of a JQF. **a**–**c**, Rabi frequency of the qubit, Rabi decay time of the qubit, and ground-state occupancy of the JQF as a function of the control amplitude represented in the square root of the photon flux $\sqrt{\dot{n}}$. The top axes show the corresponding Rabi frequency of the JQF $\Omega_{\rm f} = 2\sqrt{\gamma_{\rm ex}^{\rm f}\dot{n}}$ normalized by its external decay rate $\gamma_{\rm ex}^{\rm f}$. The black dashed lines are the analytical solutions with the qubit driven in the absence of the JQF. The blue and red solid lines are the numerically calculated results where an ideal two-level qubit and a transmon qubit with an anharmonicity of -0.1 GHz are used for the JQF, respectively. The orange dashed line shows the numerically calculated results from the approximative model with a two-level JQF. **d**–**f**, Rabi frequency, Rabi decay time, and error probability within a single Rabi cycle of the qubit as a function of the anharmonicity of the transmon-type JQF. The system parameters used in the simulations are $\gamma_{\rm ex}^{\rm q}/2\pi = 100$ kHz, $\gamma_{\rm ex}^{\rm f}/2\pi = 100$ MHz, and $\gamma_{\rm in}^{i} = \gamma_{\phi}^{i} = 0$ for $i = $ q, f.

$|{\rm g}+\rangle$) is given by $(\Omega_{\rm q}/\Omega_{\rm f})^2 = \gamma_{\rm ex}^{\rm q}/\gamma_{\rm ex}^{\rm f}$. The Rabi decay rate between $|{\rm g}+\rangle$ and $|{\rm g}-\rangle$ or between $|{\rm e}-\rangle$ and $|{\rm e}+\rangle$ is on the order of the JQF decay rate $\gamma_{\rm ex}^{\rm f}$. Thus, these additional decay rates of the qubit are on the order of the qubit decay ($\approx (\Omega_{\rm q}/\Omega_{\rm f})^2\gamma_{\rm ex}^{\rm f} = \gamma_{\rm ex}^{\rm q}$). As schematically shown in Supplementary Figure 3b, the same holds true for the Rabi oscillation between $|{\rm g}-\rangle$ and $|{\rm e}-\rangle$.

Transmon JQF versus two-level JQF

In the experiments, we use a transmon qubit as the JQF. Thus, we here consider the transmon JQF instead of the two-level JQF. The Rabi frequency of the qubit, Rabi decay time of the qubit, and ground-state occupancy of the JQF as a function of the control amplitude are numerically calculated from the rigorous model of Supplementary Equation 13, where a transmon qubit with an anharmonicity of -0.1 GHz is used for the JQF (see the red lines in Supplementary Figures 4a–c). Note that we neglect the imperfections of the qubit and the JQF as $\gamma_{\rm in}^{i} = \gamma_{\phi}^{i} = 0$ for simplicity. In the limit of large control amplitudes ($\Omega_{\rm f}/\gamma_{\rm ex}^{\rm f} \gg 1$), the Rabi decay time of the qubit with the transmon JQF is increased when compared to the two-level JQF, although there is no significant difference in the qubit Rabi frequency. As explained above, the origin of the additional qubit decay during the Rabi oscillation is the off-resonant coupling derived from the correlated decay with the lossy JQF. In the case of a transmon JQF, the JQF can be driven to be populated in higher excited states which do not have significant correlated decay with the qubit due to the large anharmonicity. Therefore, the Rabi decay time of the qubit with the transmon JQF can be closer to that without the JQF, as shown in Supplementary Figure 4b.

To further investigate the effect of the transmon-type JQF, the Rabi frequency and decay time of the qubit as a function of the anharmonicity of the transmon JQF are shown in Supplementary Figures 4d and e. The control amplitude is set to be $\sqrt{\dot{n}} = 100\ \sqrt{\text{MHz}}$. The lower anharmonicity of the JQF results in a lower Rabi frequency of the

qubit due to an effect similar to a Purcell filter. On the other hand, since a lower anharmonicity allows for populating the higher energy levels of the transmon JQF, the Rabi decay time of the qubit approaches the same value as that without a JQF. The error probability per single Rabi cycle is calculated as a product of the Rabi decay rate and the oscillation period ($\gamma^{\rm q}_{\rm Rabi} \times 2\pi/\Omega_{\rm q}$). This is plotted as a function of the anharmonicity of the transmon JQF in Supplementary Figure 4f. In the absence of the JQF, the lower limit of the error probability is given by $4\pi/3\sqrt{\gamma^{\rm q}_{\rm ex}/\dot{n}}$ (black dashed line). It is observed that the error probability is minimized when the JQF anharmonicity is close to its external coupling rate ($|\alpha_{\rm f}| \approx \gamma^{\rm f}_{\rm ex}$).

Supplementary Note 5: JQF reflection spectrum

The JQF is characterized by measuring the reflection spectrum via the control line. The JQF frequency is set to be close to that of the qubit by tuning the magnetic flux applied threading the SQUID of the JQF. Thus, we assume that $\cos(\omega_{\rm f} d) = -1$ in the model of Supplementary Note 3.

The amplitude and phase of the reflection coefficient as a function of the probe frequency and probe power are shown in Supplementary Figures 5a and b, while the cross-sections of the color plots are shown with different probe powers in Supplementary Figures 5c and d, respectively. Note that the probe-frequency step is larger than the linewidth of the qubit and that the qubit resonance is not observed in this measurement. At a smaller probe power of −146 dBm, the JQF spectrum is in the over-coupling regime, where the external coupling rate is much larger than the intrinsic loss rate, i.e. $\gamma^{\rm f}_{\rm ex} \gg \gamma^{\rm f}_{\rm in}$. The JQF transition starts to saturate around the single-photon power level, calculated as $\hbar\omega_{\rm f}(\gamma^{\rm f}_{\rm ex} + \gamma^{\rm f}_{\rm in})^2/(4\gamma^{\rm f}_{\rm ex}) \approx -120$ dBm, which would populate a linear resonator with a single photon on average. At a stronger probe power of −100 dBm, the JQF does not affect the reflection coefficient due to it being saturated.

We use the probe power dependence of the JQF spectrum to determine the system parameters of the JQF. When the first excited state is thermally populated, the transition to the second excited state can be observed in our frequency range, in principle allowing us to obtain the thermal population of the JQF. Furthermore, by fitting the reflection spectra for different powers with numerically calculated results of Supplementary Equation 26, the intrinsic loss and pure dephasing rates of the JQF can be determined independently. We use the reflection spectra with probe powers of −146 dBm, −124 dBm and −120 dBm for this characterization. Note that a phase offset and an electrical phase delay are also used as fitting parameters. The experimental results are well reproduced by numerical calculations with the optimal fitting parameters, as shown with the black lines in Figs. S5c and d. From these fits, the system parameters of the JQF are found to be $\omega_{\rm f}/2\pi = 8.0004$ GHz, $\gamma^{\rm f}_{\rm ex}/2\pi = 112$ MHz, and $\gamma^{\rm f}_{\rm in}/2\pi = 3$ MHz. The pure dephasing rate $\gamma^{\rm f}_{\phi}$ and the intrinsic thermal quanta $n^{\rm f}_{\rm th}$ are found to be too small to be reliably characterized by fitting the reflection spectra of the JQF over-coupled to the control line. We only estimate the approximate upper bounds for them by numerical simulations (Supplementary Table I).

Supplementary Note 6: Readout-resonator reflection spectrum

To characterize the thermal population of the qubit, we observe the dispersive frequency shift of the readout resonator depending on the qubit state.

The reflection spectrum of a resonator is given by

$$S^{\rm r}_{11}(\omega, \omega_{\rm r}, \kappa_{\rm ex}, \kappa_{\rm in}) = -\frac{\frac{\kappa_{\rm ex}-\kappa_{\rm in}}{2} + {\rm i}(\omega-\omega_{\rm r})}{\frac{\kappa_{\rm ex}+\kappa_{\rm in}}{2} - {\rm i}(\omega-\omega_{\rm r})}, \tag{36}$$

where ω is the probe frequency, $\omega_{\rm r}$ is the resonance frequency, $\kappa_{\rm ex}$ is the external coupling rate, and $\kappa_{\rm in}$ is the intrinsic loss rate [5].

In our setup, the readout resonator is coupled to the transmon qubit dispersively. Thus, the resonance frequency of the readout resonator is shifted by the dispersive shift χ depending on the qubit state: the resonance frequency is $\omega_{\rm r} + \chi$ ($\omega_{\rm r} - \chi$) when the qubit is in the ground (first excited) state [6].

The measurement time of the reflection signal is much longer than the time scale of the qubit dynamics in thermal equilibrium, i.e. the qubit state is hopping between the ground and first excited states during the measurement. The ratio of the dwell times of each state corresponds to the qubit thermal population. We assume that the second excited state of the qubit does not get populated. The reflection spectra of the readout resonator with the qubit in the ground and first excited states are then classically mixed as

$$S^{\rm ge}_{11}(\omega, \omega_{\rm r}, \kappa_{\rm ex}, \kappa_{\rm in}, \chi, p^{\rm q}_{\rm th}) = (1-p^{\rm q}_{\rm th})\, S^{\rm r}_{11}(\omega, \omega_{\rm r}+\chi, \kappa_{\rm ex}, \kappa_{\rm in}) + p^{\rm q}_{\rm th}\, S^{\rm r}_{11}(\omega, \omega_{\rm r}-\chi, \kappa_{\rm ex}, \kappa_{\rm in}), \tag{37}$$

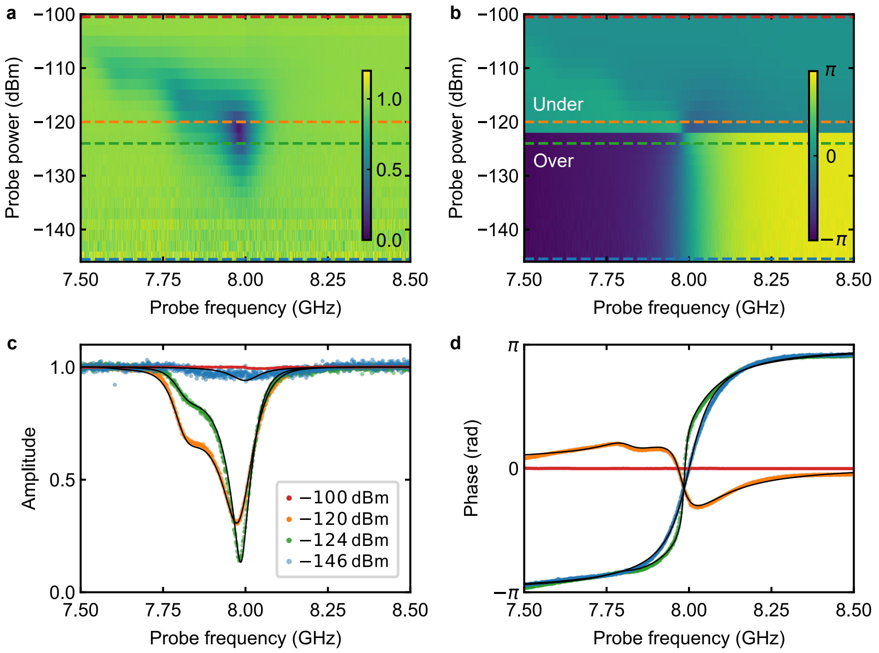

Supplementary Figure 5: Reflection spectra of JQF measured via the control line. **a,b**, Normalized amplitude and phase of the reflection coefficients of the JQF as functions of the probe frequency and power. The dashed lines indicate the probe powers of the cross-sections in **c** and **d**. Note that a phase offset is added in **b** depending on the resonance being in the over- or under-coupling regimes. **c,d**, Cross sections of **a** and **b** at different probe powers. The dots are the experimental results while the black lines are the theoretical fits for −146 dBm, −124 dBm and −120 dBm.

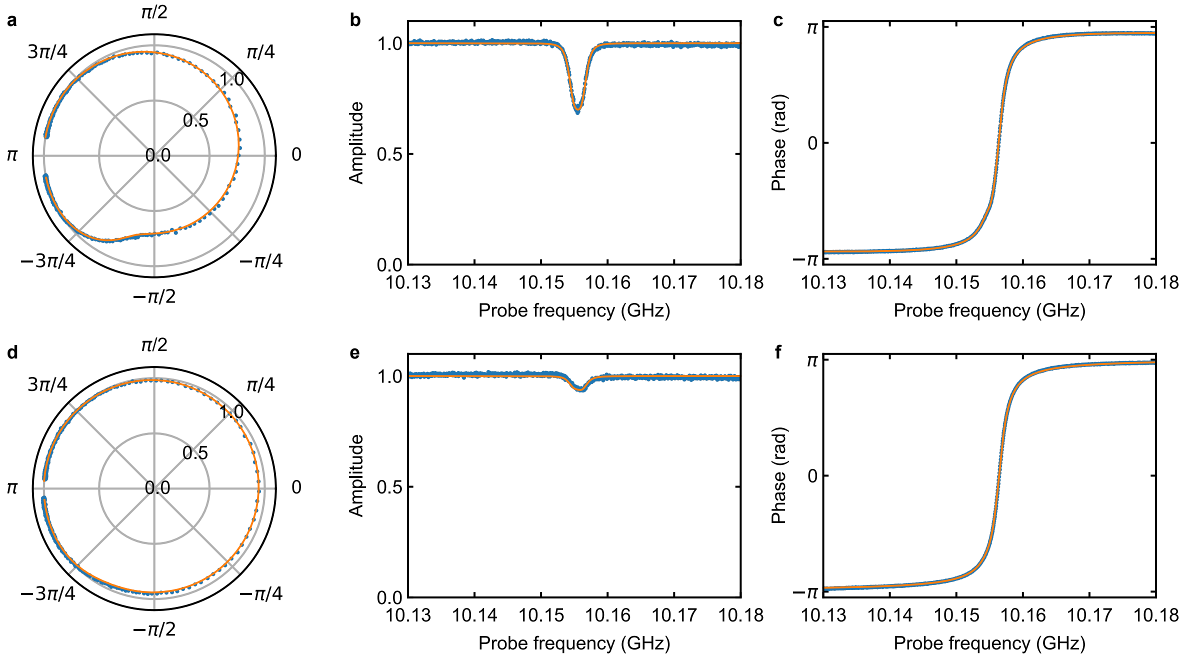

Supplementary Figure 6: Reflection spectra of the readout resonator. The dots and solid lines are the experimental results and the theoretical fits, respectively. **a**–**c**, Complex amplitude, normalized amplitude and phase of the reflection spectrum of the readout resonator when the JQF is resonant with the qubit. **d**–**f**, Same plots for the JQF out of resonance from the qubit.

where $p_{\rm th}^{\rm q}$ is the qubit thermal population in the first excited state. We use the analytical solution of the readout resonator to fit the experimental results. In this fitting, the intrinsic loss rate of the resonator and the thermal population of the qubit can be determined independently when the coupled system is in the strong dispersive regime, i.e. $\kappa_{\rm ex}+\kappa_{\rm in} \leq 2\chi$. Note that a phase offset and an electrical phase delay are also used as additional fitting parameters.

As shown in Supplementary Figures 6a–c, the complex amplitude, normalized amplitude, and phase of the reflection spectrum of the readout resonator are measured when the JQF is on resonance with the qubit, respectively. Note that the probe power is set to be −130 dBm, comparable with the single-photon power level for the readout resonator, defined as $\hbar\omega_{\rm r}(\kappa_{\rm ex}+\kappa_{\rm in})^2/(4\kappa_{\rm ex}) \approx -136$ dBm. The reflection spectrum of the readout resonator is fitted well with Supplementary Equation 37. Since we assume that the control line is well cooled down at the qubit frequency, the thermal population of the qubit is maximized due to the hotter intrinsic bath when the JQF is on resonance with the qubit and decouples the qubit from the control line. Therefore, the ratio of the reflection spectrum with the qubit in the excited state is larger, which enables us to determine the resonator frequency and dispersive shift more precisely as $\omega_{\rm r}/2\pi = 10.1564$ GHz and $2\chi/2\pi = 1.870$ MHz.

As shown in Supplementary Figures 6d–f, the reflection spectrum of the readout resonator is measured when the JQF is out of resonance from the qubit, enabling us to characterize the thermal population of the qubit in the absence of the JQF. By fitting the reflection spectrum with the determined parameters of $\omega_{\rm r}$ and χ, the external coupling and intrinsic loss rates of the resonator and the thermal population of the qubit are determined as $\kappa_{\rm ex}/2\pi = 2.152$ MHz, $\kappa_{\rm in}/2\pi = 0.015$ MHz, and $p_{\rm th}^{\rm q} = 0.028$. As shown in Supplementary Figure 9c, the thermal population of the qubit with different JQF-qubit detunings is determined in the same way. We will use the thermal population of the qubit to determine the external coupling rate of the qubit in the next section.

Supplementary Note 7: Qubit reflection spectrum

To characterize the external coupling rate of the qubit, the reflection spectrum of the qubit is measured via the control line. The external coupling rate of the qubit in the absence of the JQF is used for the calibration of the control power (see Supplementary Note 8).

Since the qubit linewidth is much smaller than its anharmonicity, under the assumption of no thermal excitation of its second excited state, the transmon qubit can be well approximated as a two-level system, i.e. the truncation number of the annihilation operator is set to 2 in the model discussed in Supplementary Note 3. Therefore, the off-diagonal element of the steady state of the master equation (Supplementary Equation 22) is analytically solved as

$$\langle 0|\hat{\rho}_{ss}|1\rangle = \mathrm{i}\frac{\sqrt{\gamma_{\rm ex}^{\rm q}\dot{n}}}{(2n_{\rm eff}^{\rm q}+1)\gamma_2^{\rm q}}\frac{1+\mathrm{i}\left(\frac{\omega-\omega_{\rm q}}{\gamma_2^{\rm q}}\right)}{1+\left(\frac{\omega-\omega_{\rm q}}{\gamma_2^{\rm q}}\right)^2+\frac{4\gamma_{\rm ex}^{\rm q}\dot{n}}{\gamma_1^{\rm q}\gamma_2^{\rm q}}}, \tag{38}$$

where $n_{\rm eff}^{\rm q} = \gamma_{\rm in}^{\rm q} n_{\rm th}^{\rm q}/(\gamma_{\rm ex}^{\rm q}+\gamma_{\rm in}^{\rm q})$ is the effective thermal quanta, $\gamma_1^{\rm q} = \gamma_{\rm ex}^{\rm q} + (2n_{\rm th}^{\rm q}+1)\gamma_{\rm in}^{\rm q}$ is the total relaxation rate, and $\gamma_2^{\rm q} = \gamma_1^{\rm q}/2 + \gamma_\phi^{\rm q}$ is the total dephasing rate [7]. Using Supplementary Equation 26, the reflection spectrum of the qubit is given by

$$S_{11}^{\rm q} = 1 - \frac{\gamma_{\rm ex}^{\rm q}}{(2n_{\rm eff}^{\rm q}+1)\gamma_2^{\rm q}}\frac{1+\mathrm{i}\left(\frac{\omega-\omega_{\rm q}}{\gamma_2^{\rm q}}\right)}{1+\left(\frac{\omega-\omega_{\rm q}}{\gamma_2^{\rm q}}\right)^2+\frac{4\gamma_{\rm ex}^{\rm q}\dot{n}}{\gamma_1^{\rm q}\gamma_2^{\rm q}}}, \tag{39}$$

where $\langle \hat{b}_{\rm q}\rangle_{\rm ss} = \langle 0|\hat{\rho}_{\rm ss}|1\rangle$ is used. In the limit of weak probe power, i.e. $\dot{n} \ll \gamma_1^{\rm q}\gamma_2^{\rm q}/(4\gamma_{\rm ex}^{\rm q})$, the reflection spectrum can be approximated as

$$S_{11}^{\rm q}(\omega, \omega_{\rm q}, \gamma_{\rm eff}^{\rm q}, \gamma_2^{\rm q}) = 1 - \frac{\gamma_{\rm eff}^{\rm q}}{\gamma_2^{\rm q} - \mathrm{i}(\omega-\omega_{\rm q})}, \tag{40}$$

where an effective rate $\gamma_{\rm eff}^{\rm q} = \gamma_{\rm ex}^{\rm q}/(2n_{\rm eff}^{\rm q}+1)$ is defined as an independent fitting parameter. Note that a phase offset and an electrical phase delay are also used as fitting parameters. Furthermore, from the experimentally-obtainable qubit thermal population $p_{\rm th}^{\rm q}$, the effective thermal quanta $n_{\rm eff}^{\rm q}$ is obtained as

$$n_{\rm eff}^{\rm q} = \frac{p_{\rm th}^{\rm q}}{1-2p_{\rm th}^{\rm q}}. \tag{41}$$

As a result, the external coupling rate of the qubit is determined to be

$$\gamma_{\rm ex}^{\rm q} = (2n_{\rm eff}^{\rm q}+1)\gamma_{\rm eff}^{\rm q}. \tag{42}$$

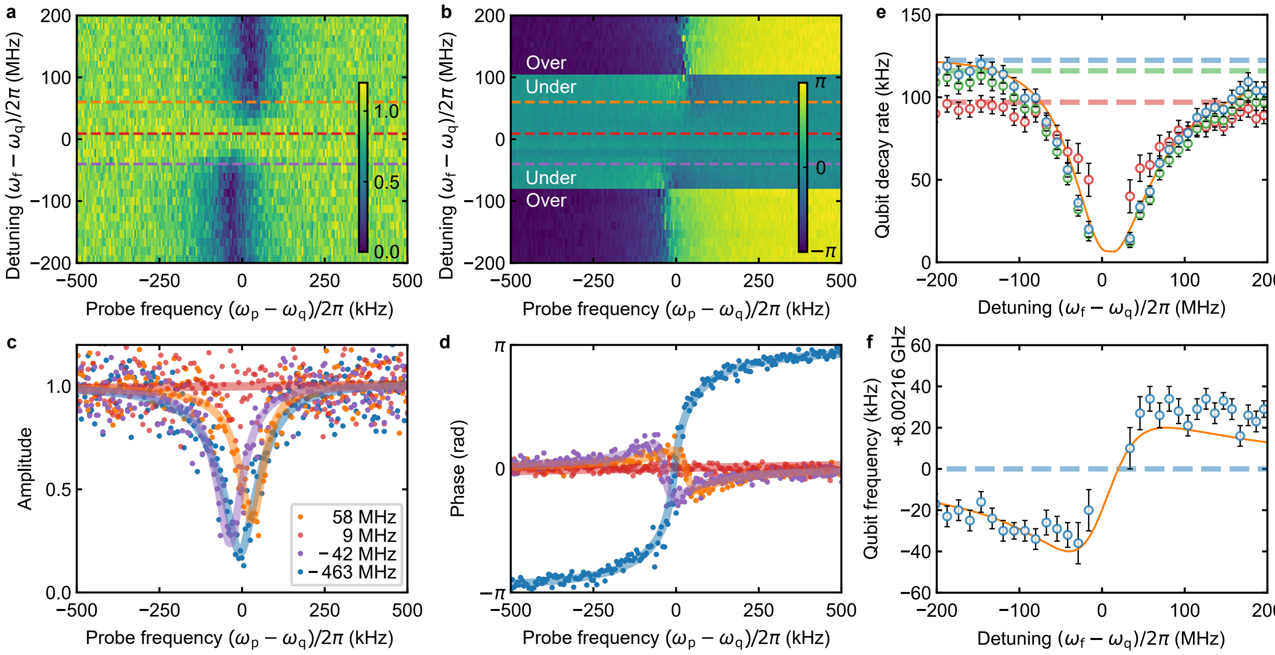

Supplementary Figure 7: Reflection spectra of the qubit measured via control line. **a**,**b**, Amplitude and phase of the reflection coefficients as functions of the probe-qubit and JQF-qubit detunings. The dashed lines indicate the detunings of the cross-sections in **c** and **d**. Note that a phase offset is added in **b** depending on the resonance being in the over- or under-coupling regimes. **c**,**d**, Cross sections of **a** and **b** at different JQF-qubit detunings. The dots and solid lines are the experimental results and the theoretical fits, respectively. **e**, Qubit decay rates as a function of the detuning. The green, red, and blue circles are the fitting results of the effective rate $\gamma^{\rm q}_{\rm eff}$, total dephasing rate $\gamma^{\rm q}_{2}$, and corrected external coupling rate $\gamma^{\rm q}_{\rm ex}$, respectively. The dashed lines depict the experimental results in the absence of the JQF. **f**, Qubit frequency as a function of the JQF-qubit detuning. The dashed line is the qubit frequency experimentally obtained in the absence of the JQF. The orange solid lines in E and F are the external coupling rate and resonance frequency of the qubit which are numerically obtained by the master equation Supplementary Equation 13, respectively The error bars represent standard errors.

The amplitude and phase of the reflection coefficient as a function of the probe frequency and the JQF-qubit detuning are shown in Supplementary Figures 7a and b, respectively. To obtain the linear response of the qubit, we set the probe power to −166 dBm in these measurements. The qubit spectrum with the far detuning from the JQF (in the absence of the JQF) is found to be in the over-coupling regime to the control line. In contrast, when the JQF is on resonance with the qubit, the signature of the qubit transition disappears, indicating that the qubit is decoupled from the control line.

As shown in Supplementary Figures 7c and d, the qubit reflection spectra with the different JQF-qubit detunings are fitted well with the theoretical model of Supplementary Equation 40. The fitting results as a function of the detuning are plotted in Supplementary Figures 7e and f. The external coupling rate of the qubit, which is determined from Supplementary Equation 42, is also shown in Supplementary Figures 7e. Note that we use the thermal population of the qubit for the various detuning, which is obtained from the numerical simulation, as shown in Supplementary Figure 9c. The full frequency bandwidth at half maximum of the suppression spectrum is found to be about 130 MHz, which roughly coincides with the external coupling rate of the JQF. The asymmetry of the suppression spectrum is explained by the non-ideal distance between the qubit and the JQF, i.e. $d = 0.526\lambda_{\rm q}$ (see Supplementary Note 9).

The numerically calculated external coupling rate and resonance frequency of the qubit as a function of the detuning are shown with the orange solid lines in Supplementary Figures 5e and f, respectively. Here, the numerical results reproduce the experimental results without any fitting parameters. The external coupling rate of the qubit in the presence of the JQF is numerically obtained by calculating the relaxation rate of the qubit with the master equation Supplementary Equation 13 where the intrinsic loss rate is set to $\gamma^{\rm q}_{\rm in} = 0$. The qubit frequency is numerically obtained by fitting the simulation results of the Ramsey sequence with a damped sinusoidal curve.

The bare external coupling rate of the qubit is used for the calibration of the qubit control power as discussed in Supplementary Note 8. When the JQF is far detuned from the qubit, the effective rate is determined as $\gamma^{\rm q}_{\rm eff}/2\pi =$ 116 kHz. Using $p^{\rm q}_{\rm th} = 0.028$ as found in Supplementary Note 6, the effective thermal quanta is determined as

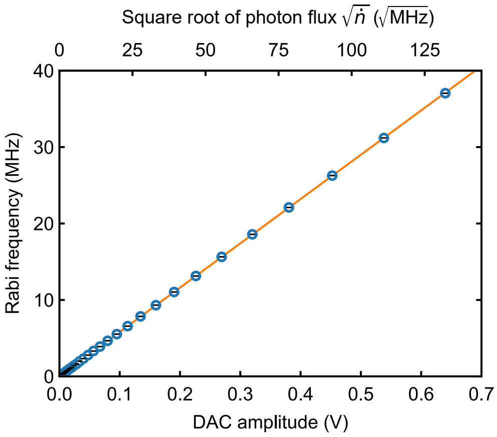

Supplementary Figure 8: Qubit Rabi frequency as a function of the control amplitude without the JQF. The dots and the solid line are the experimental results and the linear fit, respectively. The bottom and top scales describe the DAC amplitude and the calibrated control amplitude represented in the square root of the photon flux $\sqrt{\dot{n}}$, respectively. The error bars represent standard errors.

$n_{\mathrm{eff}}^{\mathrm{q}} = 0.029$. Then, the qubit external coupling rate in the absence of the JQF is determined as $\gamma_{\mathrm{ex}}^{\mathrm{q}}/2\pi = 123$ kHz.

Supplementary Note 8: Qubit Rabi oscillation

To calibrate the photon flux of the control microwave field, the qubit Rabi frequency is measured in the absence of the JQF. Rabi oscillations are observed by applying the duration-varying control pulse followed by the readout pulse with the JQF frequency set to be far detuned from the qubit frequency. By fitting the experimental results with a damped sinusoidal curve, the Rabi frequency is obtained as a function of the amplitude of the control signal generated by the digital-analog converter (DAC), as shown in Supplementary Figure 8. Usually, it is hard to precisely determine the ratio of the square root of the control photon flux to the DAC amplitude from the experimental setup. Here, this ratio is determined by comparing the slope of the Rabi frequency as a function of the DAC amplitude with $2\sqrt{\gamma_{\mathrm{ex}}^{\mathrm{q}}}$, since the Rabi frequency is given by $\Omega_{\mathrm{q}} = 2\sqrt{\gamma_{\mathrm{ex}}^{\mathrm{q}}\dot{n}}$. Using the external coupling rate of the qubit in the absence of the JQF, which is determined in Supplementary Note 7, the square root of the photon flux for a given DAC amplitude is calibrated as shown with the upper scale in Supplementary Figure 8.

Supplementary Note 9: Qubit decay times with JQF

Here, we characterize the intrinsic loss rate $\gamma_{\mathrm{in}}^{\mathrm{q}}$, pure dephasing rate $\gamma_{\phi}^{\mathrm{q}}$ and intrinsic thermal quanta $n_{\mathrm{th}}^{\mathrm{q}}$ of the qubit by measuring its relaxation and coherence as a function of the JQF-qubit detuning.

As shown in Supplementary Figure 9a, the qubit relaxation is measured as a function of the JQF-qubit detuning. By fitting the experimental results with an exponential curve, the relaxation time of the qubit is determined as shown in Supplementary Figure 9b. The thermal population of the qubit, which is obtained from the reflection spectrum of the readout resonator as discussed in Supplementary Note 6, is shown as a function of the detuning in Supplementary Figure 9c. Since not only the intrinsic loss but also the thermal population contributes to the qubit relaxation time, the intrinsic loss rate $\gamma_{\mathrm{in}}^{\mathrm{q}}$ and the intrinsic thermal quanta $n_{\mathrm{th}}^{\mathrm{q}}$ are determined simultaneously by fitting the relaxation time and thermal population of the qubit with numerical calculations based on the master equation (13). The thermal population is numerically obtained from the steady state of the master equation without a drive field. In addition, since the asymmetric shapes of the peaks in Supplementary Figures 9b and c is explained by the mismatch between the ideal distance of the half-wavelength and the actual distance, the distance d is considered as a fitting parameter. The experimental results are reproduced well by numerical simulations with optimal fitting parameters, as shown in Supplementary Figures 9b and c. The system parameters are determined as $\gamma_{\mathrm{in}}^{\mathrm{q}}/2\pi = 16$ kHz, $n_{\mathrm{th}}^{\mathrm{q}} = 0.29$, and $d = 0.526\lambda_{\mathrm{q}}$, where λ_{q} is the wavelength at the qubit frequency.

Furthermore, we measure the qubit coherence as a function of the JQF-qubit detuning. Supplementary Figure 9f shows the qubit coherence times which are determined by fitting the experimental results obtained by the Hahn-

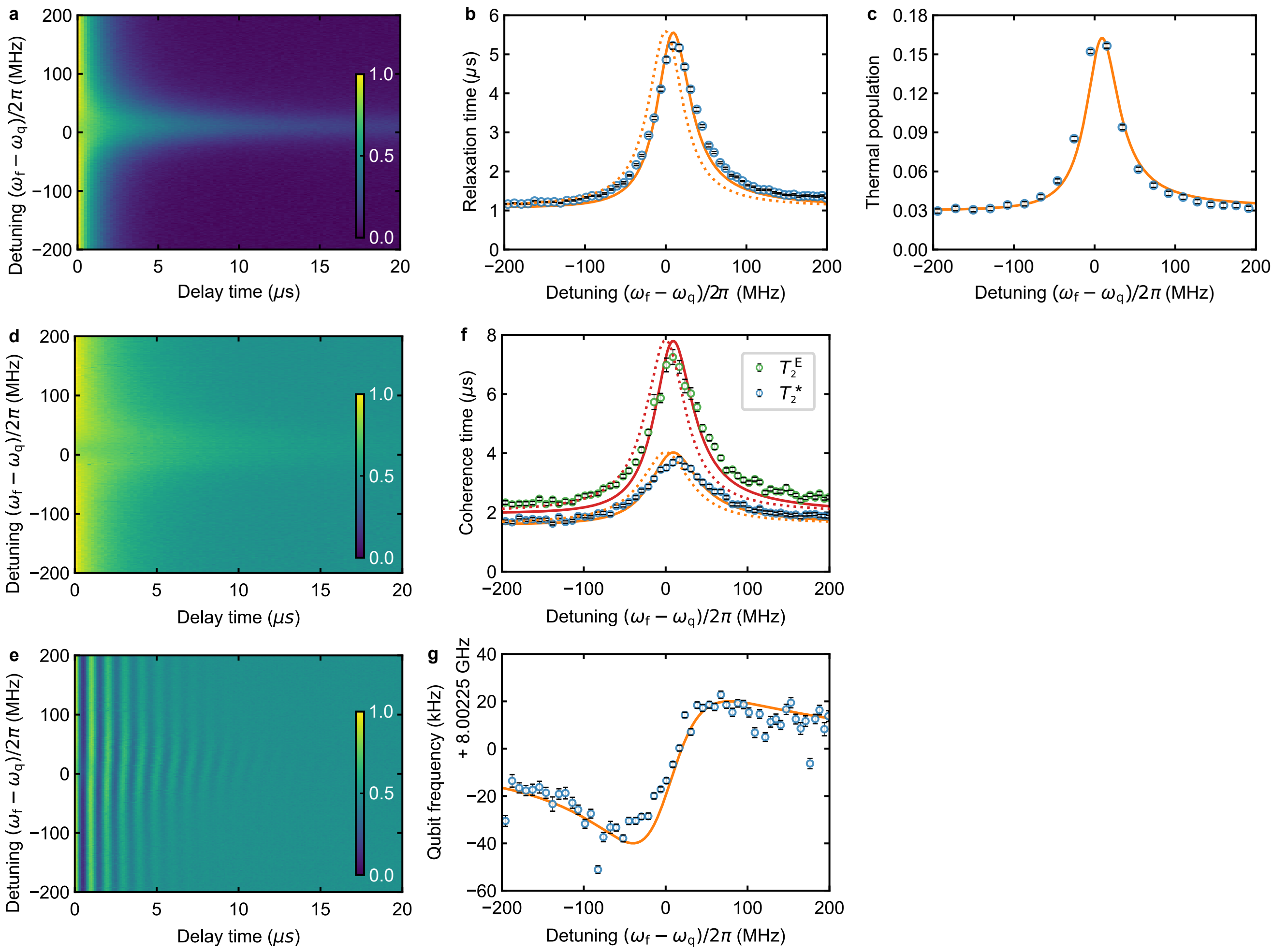

Supplementary Figure 9: Qubit relaxation, coherence and thermal population. **a**, Qubit relaxation as a function of the JQF-qubit detuning. The color scales represent the qubit populations in the excited state. **b,c**, Relaxation time and thermal population of the qubit as a function of the detuning. The circles and solid lines are the experimental results and the theoretical fits with the actual distance $d = 0.526\lambda_q$, respectively. The dotted line is the numerical simulation calculated with the ideal distance $d = 0.5\lambda_q$. **d,e**, Coherence of the qubit with the Hahn-echo and Ramsey sequences as a function of the detuning, respectively. **f,g**, Coherence times and resonance frequency of the qubit as functions of the detuning. The circles and solid lines are the experimental results and the numerical calculations, respectively. The dotted lines are the corresponding numerical calculations with the ideal distance $d = 0.5\lambda_q$. The error bars represent standard errors.

echo and Ramsey sequences (shown in Supplementary Figures 9d and e) with exponential and damped sinusoidal curves, respectively. In Supplementary Figure 9g, we show the qubit frequency which is determined from fitting the data of the Ramsey sequence with a finite detuning between the qubit frequency and the control frequency (shown in Supplementary Figures 9e). The coherence times of the qubit as a function of the detuning are fitted with the numerical calculation results obtained by the master equation (Supplementary Equation 13), where the pure dephasing rate of the qubit is considered as a fitting parameter. In the numerical simulation, the qubit is initialized in the coherent superposition state and the qubit population in the x basis is calculated after the varying delay time without a drive field. Note that the rotating frame of the numerical calculation is shifted from the bare qubit frequency by a finite detuning in order to easily distinguish the dephasing from the frequency shift of the qubit. The experimental results agree well with the numerical calculations, enabling us to determine the pure dephasing rate to be $\gamma_\phi^q/2\pi = 6$ kHz for the Hahn-echo sequence and $\gamma_\phi^q/2\pi = 25$ kHz for the Ramsey sequence, respectively. We use the pure dephasing rate from the Hahn-echo sequence for the simulation of the Rabi oscillation and the calculation of the coherence limit of the average gate error of the Clifford gates. The qubit frequency as a function of the detuning is also reproduced well by the numerical calculation, as shown in Supplementary Figure 9g.

The dotted lines in Supplementary Figures 9b and f are the decay times numerically calculated with the ideal distance $d = 0.5\lambda_{\mathrm{q}}$. Note that the same system parameters are used in these numerical simulations except for the distance. Both the results with $d = 0.526\lambda_{\mathrm{q}}$ and $d = 0.5\lambda_{\mathrm{q}}$ show the equivalent enhancements in the decay times at the different optimal JQF–qubit detuning. In other words, even if the JQF is located at a distance not exactly half a wavelength apart, with an appropriate JQF–qubit detuning we are able to enhance the qubit coherence to the level that would be achieved with the ideal distance.

[1] Hutchings, M. D. *et al.* Tunable superconducting qubits with flux-independent coherence. *Phys. Rev. Applied* **8**, 044003 (2017).

[2] Koshino, K., Kono, S. & Nakamura, Y. Protection of a Qubit via Subradiance: A Josephson Quantum Filter. *Phys. Rev. Appl.* **13**, 014051 (2020).

[3] Kono, S., Koshino, K., Tabuchi, Y., Noguchi, A. & Nakamura, Y. Quantum non-demolition detection of an itinerant microwave photon. *Nature Phys.* **14**, 546–549 (2018).

[4] Lalumière, K. *et al.* Input-output theory for waveguide qed with an ensemble of inhomogeneous atoms. *Phys. Rev. A* **88**, 043806 (2013).

[5] Clerk, A. A., Devoret, M. H., Girvin, S. M., Marquardt, F. & Schoelkopf, R. J. Introduction to quantum noise, measurement, and amplification. *Rev. Mod. Phys.* **82**, 1155 (2010).

[6] Gambetta, J. *et al.* Qubit-photon interactions in a cavity: Measurement-induced dephasing and number splitting. *Phys. Rev. A* **74**, 042318 (2006).

[7] Mirhosseini, M. *et al.* Cavity quantum electrodynamics with atom-like mirrors. *Nature* **569**, 692 (2019).
